# Dietary Methionine Restriction in Mice Elicits an Adaptive Cardiovascular Response to Hyperhomocysteinemia

**DOI:** 10.1038/srep08886

**Published:** 2015-03-06

**Authors:** Gene P. Ables, Amadou Ouattara, Thomas G. Hampton, Diana Cooke, Frantz Perodin, Ines Augie, David S. Orentreich

**Affiliations:** 1 The Orentreich Foundation for the Advancement of Science, Inc., Cold Spring-on-Hudson, NY; 2 Mouse Specifics, Inc., Quincy, MA

## Abstract

Dietary methionine restriction (MR) in rodents increased lifespan despite higher heart-to-body weight ratio (w/w) and hyperhomocysteinemia, which are symptoms associated with increased risk for cardiovascular disease. We investigated this paradoxical effect of MR on cardiac function using young, old, and apolipoprotein E-deficient (ApoE-KO) mice. Indeed, MR animals exhibited higher heart-to-body weight ratio (w/w) and hyperhomocysteinemia with a molecular pattern consistent with cardiac stress while maintaining the integrity of cardiac structure. Baseline cardiac function, which was measured by non-invasive electrocardiography (ECG), showed that young MR mice had prolonged QRS intervals compared with control-fed (CF) mice, whereas old and ApoE-KO mice showed similar results for both groups. Following β-adrenergic challenge, responses of MR mice were either similar or attenuated compared with CF mice. Cardiac contractility, which was measured by isolated heart retrograde perfusion, was similar in both groups of old mice. Finally, the MR diet induced secretion of cardioprotective hormones, adiponectin and fibroblast growth factor 21 (FGF21), in MR mice with concomitant alterations in cardiac metabolic molecular signatures. Our findings demonstrate that MR diet does not alter cardiac function in mice despite the presence of hyperhomocysteinemia because of the adaptive responses of increased adiponectin and FGF21 levels.

Dietary methionine restriction (MR) has been demonstrated to extend the lifespan of rodents, drosophila, nematodes, and yeasts^1^,^2^,^3^,^4^,^5^. Rats fed an 80% reduced methionine diet exhibited a 40% increase in median and maximum lifespan compared with their control-fed (CF) counterparts^2^,^3^. In mice, lifespan is extended when MR is initiated at young and middle ages^1^,^6^. Although the descriptions of the mortalities are insufficient, it was suggested that the extension of the lifespan could be a result of delayed tumor formation^1^. Interestingly, despite their increased lifespan, MR-fed rodents also exhibit higher heart-to-body weight ratio (w/w) and hyperhomocysteinemia, which are symptoms associated with increased risk of cardiovascular disease^2^,^7^,^8^. Because of the paradoxical effects of MR on homocysteine and lifespan, we investigated its effects on mouse cardiac function.

Hyperhomocysteinemia is an independent risk factor for cardiovascular disease^9^. Homocysteine is generated from methionine (Met) through the successive enzymatic activations of S-adenosylmethionine and S-adenosyl-homocysteine^10^. Once generated, homocysteine is either metabolized by cystathionine β-synthase (CBS) to cysteine via the transsulfuration pathway or remethylated to Met by 5,10-methylenetetrahydrofolate reductase or betaine homocysteine methyltransferase^11^. Severe arteriosclerosis with hyperhomocysteinemia was first reported in association with a rare genetic condition in humans, which is also characterized by abnormal cobalamine metabolism and CBS deficiency^12^. Since then, homocysteine has been strongly correlated with vascular dysfunction^13^,^14^. Vascular damage caused by homocysteine could be a result of proinflammatory factors, oxidative stress, and endoplasmic reticulum stress^15^. Recent reports have implicated p53 DNA hypomethylation in cardiac injury^16^. However, in clinical settings, homocysteine-lowering interventions have been suggested to be ineffective in lowering cardiovascular risk^17^,^18^,^19^,^20^. Thus, the specific role of homocysteine on cardiovascular disease remains unclear.

Our study showed that MR increased the heart-to-body weight ratio (w/w) and induced hyperhomocysteinemia in young, old, and apolipoprotein E KO (ApoE-KO) mice. The cardiac functions in rodent models were not affected by MR as measured by non-invasive electrocardiography (ECG) in conscious mice. Cardiac contractility was also similar between old CF and MR mice regardless of calcium or isoproterenol challenge. Dietary MR increased the plasma levels of adiponectin and fibroblast growth factor 21 (FGF21), hormones that confer cardioprotection. Overall, our study suggests that the MR diet did not alter cardiac function despite the presence of hyperhomocysteinemia in mice because of an adaptive response of cardioprotective hormones.

## Results

### Young MR mice exhibited predisposition to cardiovascular disease

**Figure 1:**
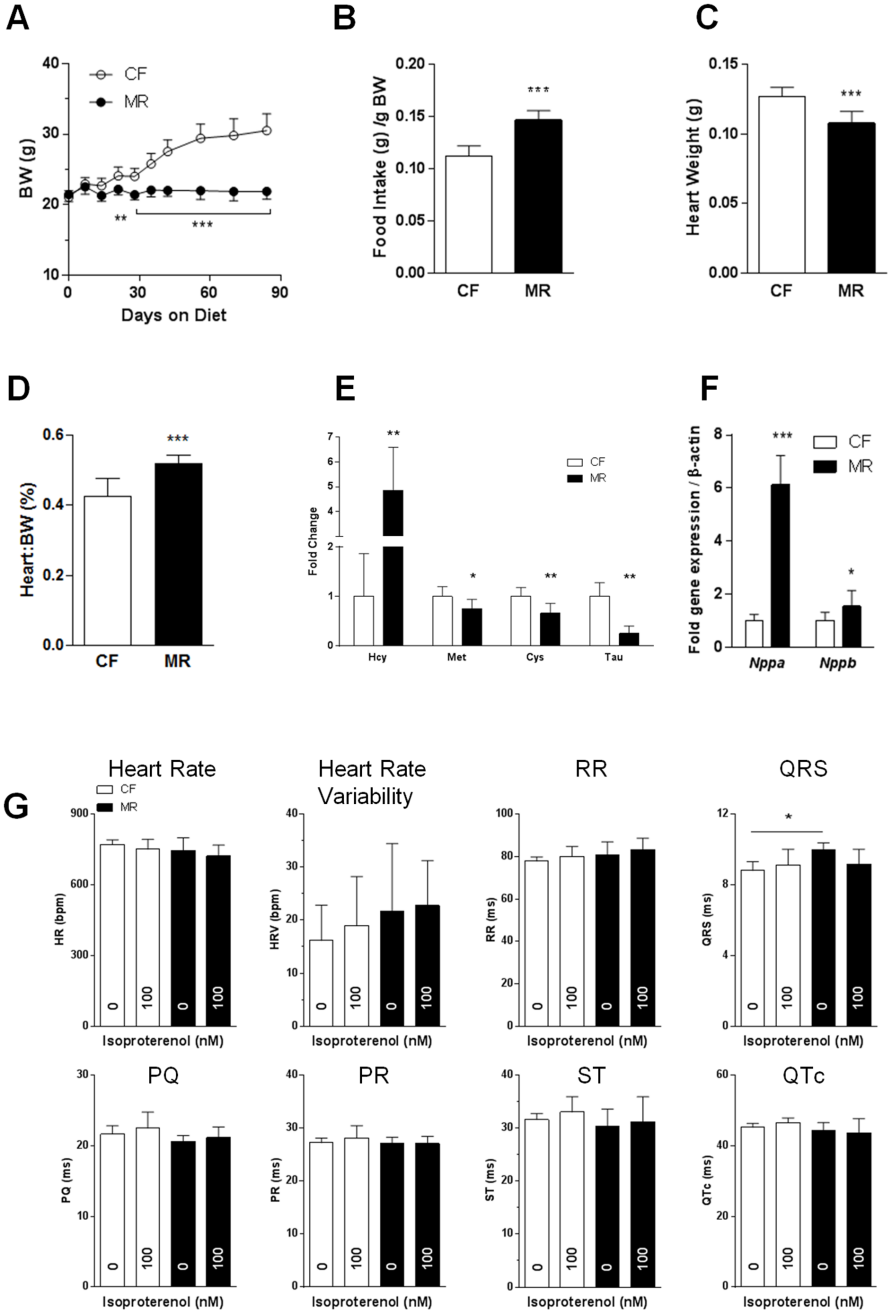
Young MR mice exhibit a predisposition to cardiovascular disease. (A). Body weight of 8-week-old mice under the CF (0.84% methionine) or MR (0.12% methionine) diets for 12 weeks. (B). Food intake of the mice per gram body weight. (C). and (D). Heart weight and heart-to-body weight ratios of the mice upon sacrifice. (E). Fold changes of sulfur amino acids in the plasma of MR mice relative to CF mice based on HPLC measurements. (F). Cardiac gene expression as measured by quantitative real-time PCR using TaqMan primers for *Nppa* and *Nppb*. (G). Non-invasive ECGs performed on conscious mice at basal conditions and following daily repeated 100 nM isoproterenol injections. Data were analyzed using a 2-way (A) or 1-way (G) ANOVA with Bonferroni post-tests or Student's unpaired *t*-tests (B–F) (n = 7–8 per group, *P < 0.05, **P < 0.01, ***P < 0.001).

Young MR mice are characterized by low weight gain despite hyperphagia^7^. Previous studies have shown that MR rodents have higher heart-to-body weight ratios^2^,^7^ and hyperhomocysteinemia^8^, well-known risk factors for cardiovascular disease; therefore, we assessed whether MR affects cardiac function. We used young mice, 8 weeks old, that were put on the diet for 12 weeks in our experiments. Our results indicate that young MR mice had lower body weights with hyperphagia, decreased heart weights, and higher heart-to-body weight ratio (w/w) compared with young CF mice (Figures 1A–1D). Immunoassay analysis revealed that plasma homocysteine levels were increased by 83% in young MR mice compared with CF mice (13.2 ± 3.2 μmol/l vs. 7.2 ± 0.6 μmol/l, n = 7 per group, P < 0.01). A more stringent amino acid analysis by high-performance liquid chromatography (HPLC) showed that plasma homocysteine levels were increased by 5-fold (P < 0.01) in young MR mice compared to young CF mice, whereas the other sulfur amino acids (methionine, cysteine, and taurine) decreased by 25%–70% (P < 0.05–P < 0.01, Figure 1E). Cardiac gene expression analysis in young MR mice showed an upregulation of the cardiac hypertrophy markers *Nppa* and *Nppb* compared with young CF mice (Figure 1F). Histological analyses of the cardiac tissues showed similar cardiomyocyte sizes and Ki67-stained cells in both groups (Supplemental Figure 1).

We next determined whether MR affected cardiac function using non-invasive ECG in conscious mice as previously described^21^,^22^. At basal conditions, we found a 12% (P < 0.05) increase in the QRS interval in young MR mice compared with young CF mice, whereas heart rate (HR), heart rate variability (HRV), and RR, PQ, PR, ST, and QT_c_ intervals were similar between the 2 groups (Figure 1G). To examine the effects of MR on cardiac function under stress conditions, we induced acute cardiac hypertrophy by daily repeated β-adrenergic stimulation using isoproterenol. We found that both groups had similar responses as measured by ECG, suggesting that young mice were resistant to acute stimulation (Figure 1G). To test time-course effects of MR on cardiac function at basal conditions, we used 8 week old mice that were either on acute (3 weeks) or chronic (36 weeks) MR diets. Based on non-invasive ECG, cardiac parameters were similar in both groups after acute feeding. Chronic feeding lowered HR by 5% (P < 0.05) in MR mice, which corresponded to 5% (P < 0.05) longer RR segment compared to CF counterparts (Supplementary Figures 3 and 4). Taken together, these data suggest that MR may predispose young mice to the development of cardiovascular disease.

### The MR diet predisposed old mice to cardiovascular disease but did not alter cardiac function

**Figure 2:**
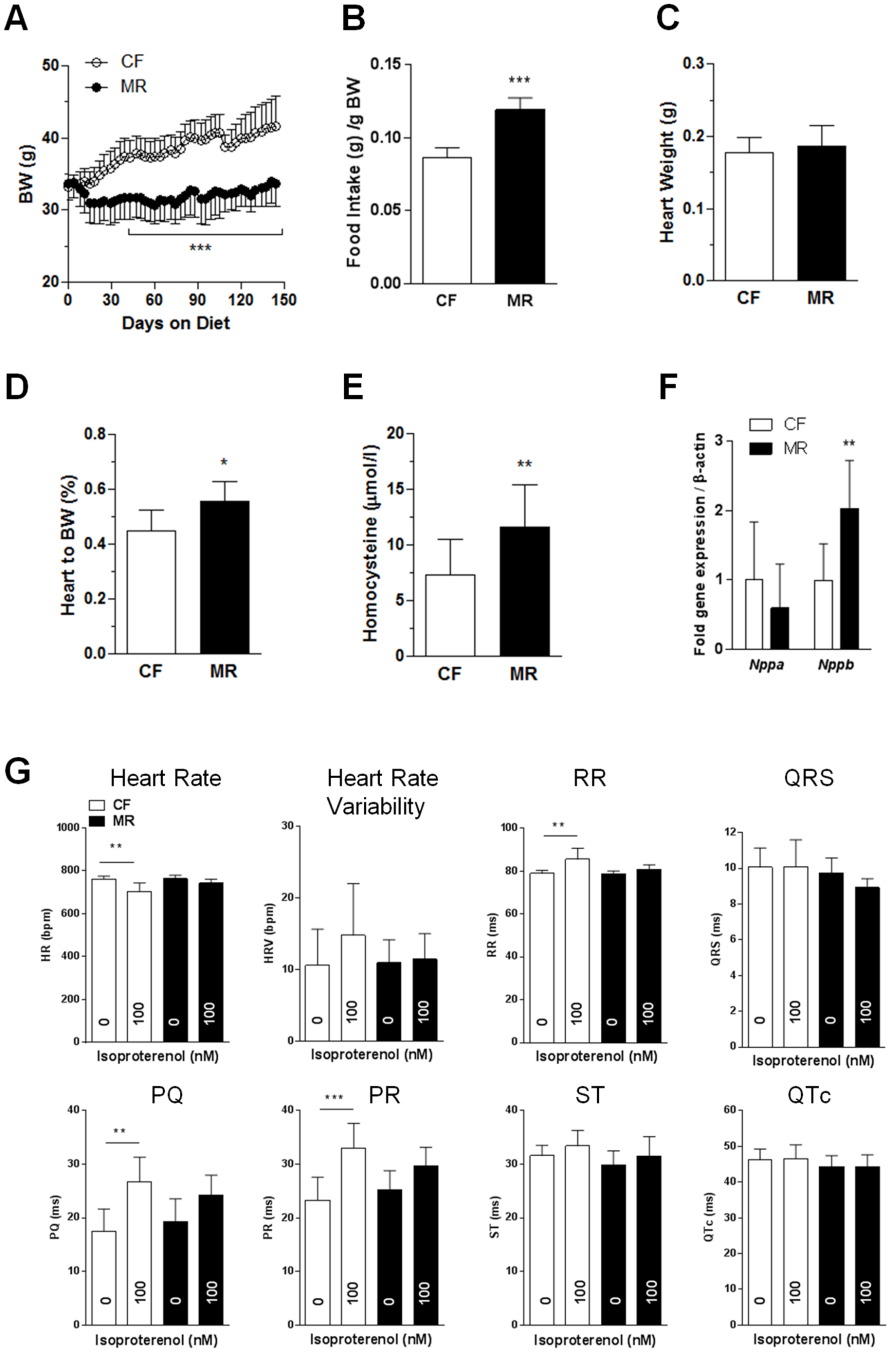
The MR diet predisposed old mice to cardiovascular disease but did not alter cardiac function. (A). Body weight of the 60-week-old mice under the CF (0.84% methionine) or MR (0.12% methionine) diets for 14 weeks. (B). Food intake of the mice per gram body weight. (C). and (D). Heart weight and heart-to-body weight ratios of the mice upon sacrifice. (E). Plasma homocysteine levels as measured by immunoassay. (F). Cardiac gene expression as measured by quantitative real-time PCR using TaqMan primers for *Nppa* and *Nppb*. (G). Non-invasive ECG performed on conscious mice at basal conditions and following daily repeated 100 nM isoproterenol injections. Data were analyzed by 2-way (A) or 1-way (G) ANOVA with Bonferroni post-tests or Student's unpaired *t*-tests (B–F) (n = 7 per group, *P < 0.05, **P < 0.01, ***P < 0.001).

We next examined old mice to determine whether the MR diet had an age-related effect on cardiac function. For this experiment, diets were initiated in 60-week-old mice for 14 weeks. Our data indicate that old MR mice had lower body weights with hyperphagia, similar absolute heart weights, and higher heart-to-body weight ratio (w/w) compared with old CF mice (Figures 2A–2D). Old MR mice also showed a 36% (P < 0.01) increase in plasma homocysteine levels compared with old CF mice (Figure 2E) when measured using immunoassay. Cardiac gene expression analysis in old MR mice showed an upregulation of the cardiac hypertrophy marker *Nppb*, but not *Nppa*, compared with old CF mice (Figure 2F). Histological analyses of the cardiac tissues showed that MR mice had smaller cardiomyocytes compared with the CF group, whereas the immunohistochemistry staining results for Ki67 were similar for both groups (Supplemental Figure 2).

Non-invasive ECG experiments in conscious mice were conducted to determine whether MR would affect cardiac function in old mice. The measurable parameters in old mice on both diets were similar at basal conditions (Figure 2G). We then assessed whether MR affects the cardiac function in older mice under stress conditions by daily repeated β-adrenergic administration using isoproterenol. Following stimulation, old CF mice exhibited a decrease of 7% in HR (P < 0.001) and an increase of 8% in RR (P < 0.001), 53% in PQ (P < 0.01), and 42% in PR (P < 0.001) segments relative to basal levels, whereas old MR mice did not respond to the stimulation (Figure 2G). These data suggest that MR in old mice has an attenuated response to sympathetic stimulation.

We then investigated cardiac contractility in old mice by retrograde isolated heart perfusion and found no differences in the HRs at basal conditions between both groups. To test whether MR affected contractility under stress conditions, their hearts were challenged with Ca^2+^ and isoproterenol. Relative to basal conditions, the degree of response following stimulation was similar in old CF and MR mice based on HR, peak systolic pressure (mean PSP), end diastolic pressure (mean EDP), left ventricular developed systolic pressure (devP), myocardial contractility (dP/dT_max_), and diastolic relaxation (dP/dT_min_) measurements (Table 1). Overall, these data suggest that MR in old mice did not affect cardiac function or contractility despite their predisposition to cardiovascular disease.

### MR in ApoE-KO mice attenuated the effects on cardiac function following β-adrenergic stimulation

**Figure 3:**
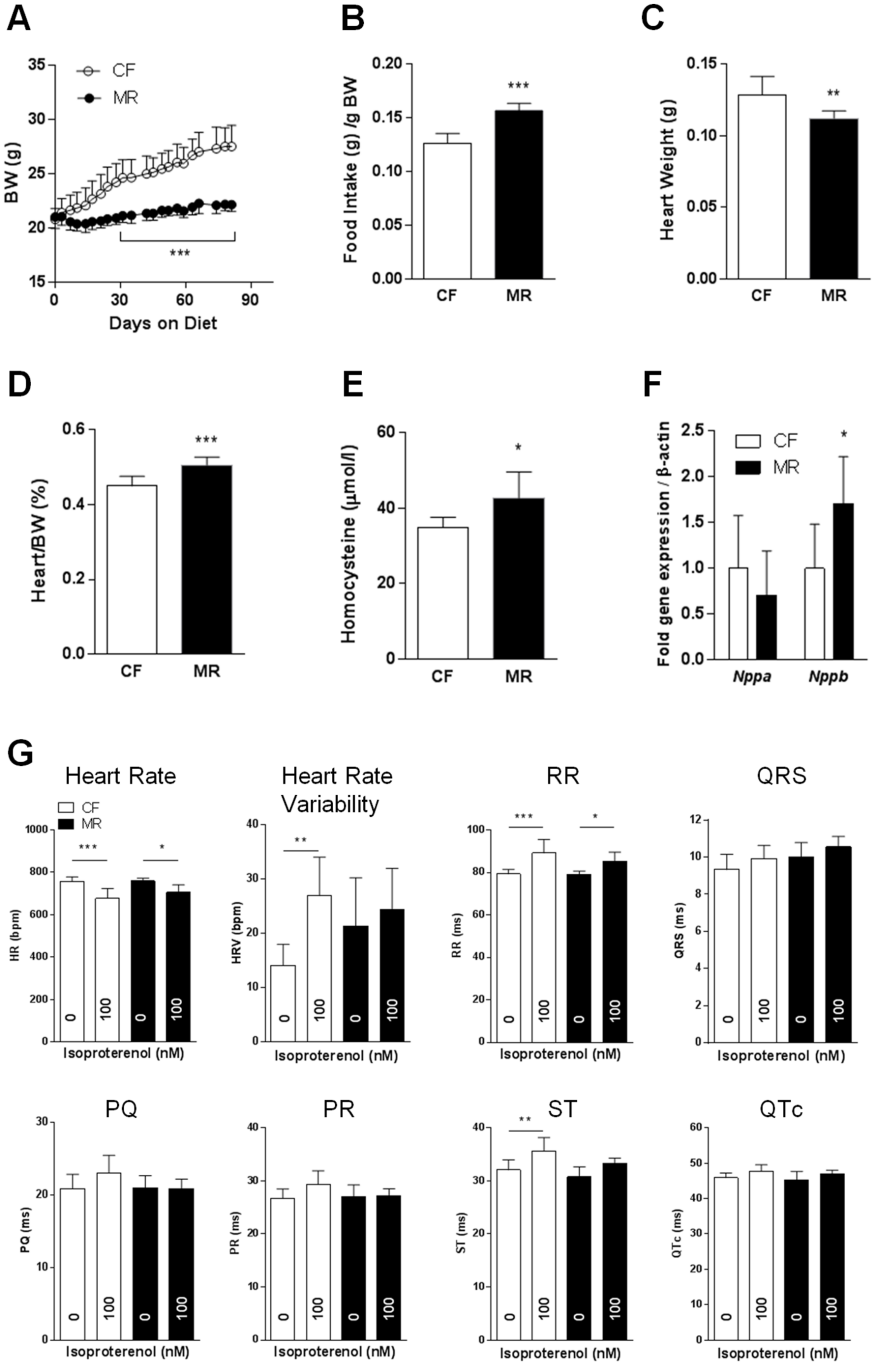
MR in ApoE-KO mice attenuated the effects of β-adrenergic stimulation on cardiac function. (A). Body weight of 8-week-old ApoE-KO mice given CF (0.84% methionine) or MR (0.12% methionine) diets for 12 weeks. (B). Food intake of the mice per gram body weight. (C). and (D). Heart weight and heart-to-body weight ratios of the mice upon sacrifice. (E). Plasma homocysteine levels as measured by immunoassay. (F). Cardiac gene expression as measured by quantitative real-time PCR using TaqMan primers for *Nppa* and *Nppb*. (G). Non-invasive ECG performed on conscious mice at basal conditions and following daily repeated 100 nM isoproterenol injections. Data were analyzed by 2-way (A) or 1-way (G) ANOVA with Bonferroni post-tests or Student's unpaired *t*-tests (B–F) (n = 7 per group, *P < 0.05, **P < 0.01, ***P < 0.001).

Because Met-induced hyperhomocysteinemia is observed in the atherosclerosis model ApoE-KO mice^23^,^24^,^25^, we examined whether MR-induced hyperhomocysteinemia affects cardiac function in these mice. We used 8-week-old mice on the diet for 12 weeks. Consistent with other models used in this study, ApoE-KO MR mice had lower body weight with hyperphagia, decreased absolute heart weight, and higher heart-to-body weight ratio (w/w) as well as hyperhomocysteinemia compared with ApoE-KO CF mice (Figures 3A–3E). Cardiac gene expression analysis showed an upregulation of the cardiac hypertrophy marker *Nppb*, but not *Nppa*, in ApoE-KO MR mice compared with ApoE-KO CF mice (Figure 3F).

We next examined the effects of MR on ApoE-KO mice by performing non-invasive ECG in conscious mice. At basal conditions, all measurable parameters were similar in ApoE-KO mice fed CF and MR diets. The induction of acute cardiac hypertrophy by daily repeated β-adrenergic administration of isoproterenol showed that ApoE-KO CF mice had a 12% decrease in HR (P < 0.001 vs. CF baseline) with a 12% increase in RR (P < 0.001 vs. CF baseline) and 11% increase in ST (P < 0.01 vs. CF baseline) intervals compared with ApoE-KO MR mice (Figure 3G). However, the response of ApoE-KO MR mice to isoproterenol was not as robust as that of ApoE-KO CF mice; ApoE-KO MR mice showed a 7% decrease in HR (P < 0.05 vs. MR baseline) with an 8% increase in RR interval (P < 0.05 vs. MR baseline) (Figure 3G). These data suggest that MR did not affect ApoE-KO cardiac function at basal conditions and attenuated the response to β-adrenergic stimulation. Taken together, our results indicate that MR in ApoE-KO mice was associated with a modest stimulatory response despite predisposition to cardiovascular disease.

### Dietary MR increased adiponectin and FGF21 levels and altered cardiac metabolic signaling

**Figure 4:**
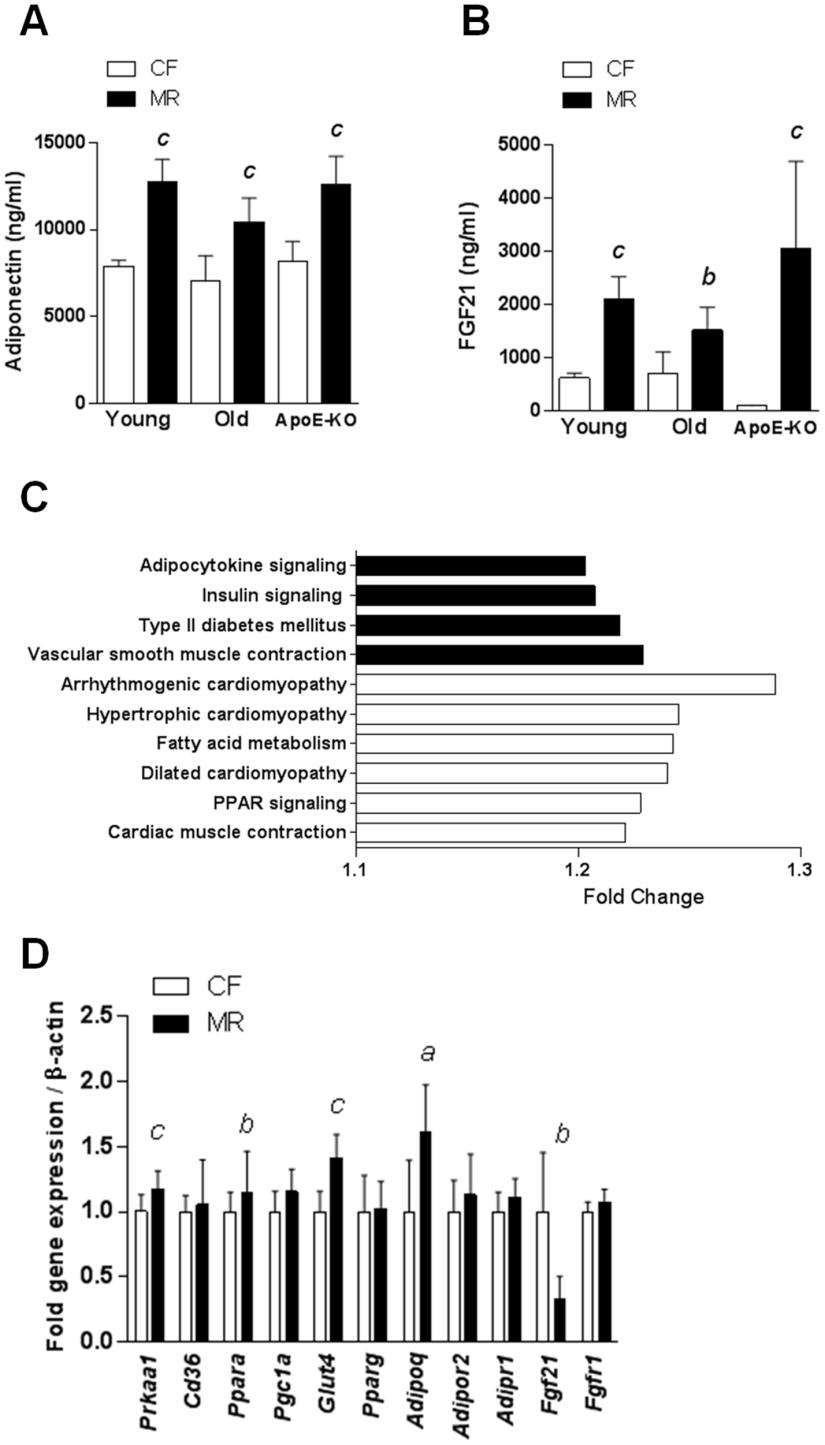
Dietary MR increased the adiponectin and FGF21 levels and altered cardiac metabolic signaling. Plasma adiponectin (A) and FGF21 (B) levels in young, old, and ApoE-KO mice as determined by ELISA. (C). Gene set enrichment analysis of canonical pathways affected in the hearts of the MR mice compared with their CF counterparts (n = 4 per group) based on the Kyoto Encyclopedia of Genes and Genomes database. Upregulated (black bars) and downregulated (clear bars) pathways were altered by ≥1.2-fold and had a false discovery rate of q < 0.01 and significance of P < 0.001. (D). Cardiac gene expression analysis in CF and MR mice using quantitative real-time PCR with TaqMan primers. Data were analyzed by Student's unpaired *t*-test relative to the control groups in (A), (B), and (D) (n = 7–8 per group, *^a^P* < 0.05, *^b^P* < 0.01, *^c^P* < 0.001).

We then assessed the mechanisms underlying the modest changes in cardiac function in MR mice despite their increased risk of cardiovascular disease. In general, MR mice are characterized by increased levels of adiponectin and FGF21^7^,^26^,^27^,^28^, two hormones that confer cardioprotection^29^,^30^. Consistent with previous studies, we showed that adiponectin and FGF21 were increased in young, old, and ApoE-KO mice (Figures 4A and 4B). In addition, both hormones were also higher in both groups of acute and chronic MR mice compared to CF (Supplementary Figures 3 and 4, respectively). To identify the MR-affected molecular pathways in mouse hearts, gene set enrichment analysis was conducted for young mice using the Kyoto Encyclopedia of Genes and Genomes database (Figure 4C). We selected the canonical pathways that were either upregulated or downregulated ≥ 1.2-fold and applied a false discovery rate of q < 0.01 with a significance of P < 0.001. Upregulated canonical pathways included those related to adipocytokine, insulin, and type 2 diabetes signaling (e.g., *Adipor1*, *Prkab1*, and *Acacb*). Downregulated canonical pathways included those involved in arrhythmogenic, hypertrophic, and dilated cardiomyopathy signaling (e.g., *Itgb6*, *Cacna1s*, and *Hadh*), fatty acid metabolism, and peroxisome proliferator-activated receptor (PPAR) signaling (e.g., *Acaa2*, *Cpt2*, and *Acsl6*) (Supplemental Table 1). Quantitative real-time PCR analysis revealed that the MR diet affected cardiac metabolic signaling, indicated by the upregulation of *Prkaa1* and *Glut4*; however, *Cd36*, *Ppara*, *Pgc1a*, and *Pparg* were not affected (Figure 4D). In addition, gene expression of *Adipoq* was upregulated and *Fgf21* was downregulated in the cardiac tissues of the MR mice; however, expression of the corresponding receptors *Adipor1*, *Adipor2*, and *Fgfr1* were similar in both groups (Figure 4D). Taken together, increased adiponectin and FGF21 hormone levels in MR mice may explain, at least in part, the effects of MR on cardiac function.

## Discussion

We conducted this study to determine the effects of MR on cardiac function in mice with concomitant hyperhomocysteinemia. Homocysteine is an established risk factor for cardiovascular disease^12^,^31^. Our results are consistent with previous MR studies that have suggested that hyperhomocysteinemia may result from inhibition of the homocysteine transsulfuration pathway, which is shown by the reduced Met, Cys, and taurine levels^8^ and decreased hepatic protein expression of the CBS enzyme^32^. In addition, we showed that young, old, and ApoE-KO MR mice developed hyperhomocysteinemia with higher heart-to-body weight ratio (w/w), which is consistent with previous MR studies^2^,^7^,^8^. Furthermore, MR mice exhibited molecular signatures consistent with cardiac stress. Interestingly, we did not observe any effects of MR on the integrity of mouse cardiac structures based on histology. Finally, based on non-invasive ECG and *ex vivo* experiments, the response of MR mice to β-adrenergic stimulation was similar or attenuated compared with those of CF mice. To our knowledge, this is the first study to show that MR may not affect cardiac function in mice despite hyperhomocysteinemia.

The cardiovascular effects of homocysteine remain unclear. Hyperhomocysteinemia has been implicated in vascular damage in CBS-deficient mice that developed vascular remodeling, cardiomyocyte dysfunction, and increased apoptosis^33^,^34^. Similarly, mice that were fed high-fat, high-Met diets developed early and accelerated atherosclerosis^35^. In contrast, mice that were deficient in either CBS or 5,10-methylenetetrahydrofolate reductase developed hyperhomocysteinemia and endothelial dysfunction without atherosclerotic lesions^36^,^37^. Moreover, mice with Met-induced hyperhomocysteinemia that were fed chow or a Western-type diet did not develop atherosclerosis^38^. Finally, clinical trials have shown that lowering homocysteine levels by administering vitamins and folic acid does not lead to substantial beneficial vascular outcomes^39^,^40^. Therefore, the specific effect of homocysteine on cardiovascular disease remains to be determined.

In this study, hyperhomocysteinemia and higher heart-to-body weight ratio (w/w) were evident in young, old, and ApoE-KO MR mice; however, cardiac function and responses to β-adrenergic stimulation varied according to non-invasive ECG tests performed on conscious mice. At basal conditions, young and chronic-fed MR mice exhibited signs of cardiac hypertrophy, whereas old and ApoE-KO mice did not show differences in any of the ECG parameters. Upon β-adrenergic stimulation, CF mice responded with extended ECG segments, whereas the MR mice exhibited mild sensitivities to isoproterenol stimulation. Previous studies have shown that hearts of old mice begin to develop left ventricular systolic dysfunction at ~18 months of age^41^. Additionally, mice fed a homocysteine-enriched diet showed prolonged QRS, QTc, and PR intervals and decreased left ventricular performance as a result of cardiac remodeling by the matrix metalloproteinases^42^. Furthermore, direct acute administration of different isoforms of homocysteine to isolated rat hearts affected contractility and reduced coronary flow^43^. We found that MR in 72-week-old mice did not affect cardiac contractility through measurements by retrograde cardiac perfusion following Ca^2+^ and isoproterenol stimulations. Although our values differ from published reports using Langendorff tests^44^, our data are within range of a previous study that used a similar procedure^45^. Also, the perfusate buffer was maintained at 32°C to preserve intracellular calcium integrity, as reported previously^46^. Overall, our results suggest that MR initiated later in life confers modest effects on cardiac function and does not affect cardiac contractility. It would be interesting to conduct echocardiography experiments to further characterize the effects of MR on cardiac function in mice.

Met-supplemented and vitamin B-deficient diets fed to ApoE-KO mice to induce hyperhomocysteinemia have been shown to promote early atherosclerosis and plaque fibrosis, but they do not induce plaque rupture^25^. Additionally, hyperhomocysteinemia in ApoE-KO mice is associated with altered lipid metabolism without the progression of atherosclerotic lesions^47^. However, when hyperhomocysteinemic ApoE-KO mice were fed Western-type or atherogenic diets, they exhibited an accelerated development of atherosclerotic lesions^38^, which may be a result of enhanced p53 signaling^48^. Interestingly, the vitamin B supplementation of hyperhomocysteinemic ApoE-KO mice appears to promote homocysteine-independent protection against atherosclerosis^49^. We revealed that the cardiac function of ApoE-KO MR mice were modestly affected following β-adrenegric stimulation compared with ApoE-KO CF mice. Whether MR-induced hyperhomocysteinemia causes vascular damage in ApoE-KO mice remains to be investigated.

We emphasize the pleiotropic effects of MR and its possible effect on cardiovascular function despite the presence of hyperhomocysteinemia. Our data are consistent with previous studies that showed increased levels of adiponectin and FGF21^7^,^26^,^27^,^28^,^50^, two hormones that confer cardioprotection^29^,^30^, in MR mice.

FGF21 is mainly secreted by the liver^51^ and has been suggested to exert cardioprotective effects via the FGFR1/β-Klotho-PI3K-Akt1-BAD signaling network in a mouse model for myocardial ischemia^29^. Additionally, studies of macrophage foam cells have demonstrated that FGF21 can promote cholesterol efflux by upregulating ABCA1 through the ERK1/2-PPARγ-LXRα pathway^52^. Additionally, peroxisome proliferator-activated receptor gamma coactivator 1-alpha (PGC1α) has been implicated in cardioprotection via FGF21^53^. Although our data showed increased plasma FGF21 hormone levels in MR mice, the decreased cardiac expression of the *Fgf21* gene observed in the young MR mice and lack of alterations in the expressions of its receptor *Ffgfr1* and downstream targets *Ppara* and *Pgc1a* suggest the presence of alternative cardioprotective mechanisms in the MR model.

Adiponectin is mainly secreted from adipose tissue^54^ and protects the heart from ischemia-reperfusion injury through both AMP-activated protein kinase (AMPK) and cyclooxygenase (COX)-2-dependent mechanisms^55^. Additionally, it has been demonstrated in primary cardiomyocytes and isolated working hearts that adiponectin improves cardiac metabolism via AMPK and the adaptor protein-containing pleckstrin homology domain, phosphotyrosine-binding domain, and Leu zipper motif (APPL1)^56^. Our data in MR mice showed an increase in plasma adiponectin levels, upregulation of the adipocytokine signaling pathway, and increase in the gene expression of *Adipoq* and its downstream targets, *Glut4* and *Prkaa1*, suggesting that MR possibly exerts its cardioprotective effects via the adiponectin signaling pathway.

The possible protective effects of these hormones in MR mice were supported, at least in part, by the gene set enrichment analysis. In this study, the cardiac genetic profiles of MR mice suggested that they possessed increased risks for cardiovascular disease. Interestingly, the pathway analysis of the genes expressed in hearts of MR mice showed an upregulation of adipocytokines and insulin signaling, whereas cardiomyopathy pathways were downregulated. Further investigations on the cardiac-specific effects of FGF21 and adiponectin under MR conditions would provide increased insight into the possible underlying molecular mechanisms.

Overall, our study highlights the importance of whole-organism physiology and homeostasis. The finding that MR extended lifespan concomitant with hyperhomocysteinemia is inconsistent with reported data that have correlated homocysteine levels with cardiovascular disease. Here, we report that MR induces hyperhomocysteinemia without affecting cardiac function, which is possibly a result of compensatory mechanisms elicited by the hepatic and adipose tissue secretions of the cardioprotective hormones FGF21 and adiponectin, respectively.

## Methods

### Animal care

All animal experiments were carried out with the approved guidelines of the Institutional Animal Care and Use Committee of the Orentreich Foundation for the Advancement of Science, Inc., and conducted following the 8^th^ edition of the National Research Council guidelines for laboratory animal use (Permit Number: 0511MB). Male C57BL/6J (stock #000664) and ApoE-KO (B6.129P2-Apoe^tm1Unc^/J, stock #002052) mice were purchased from the Jackson Laboratories (Bar Harbor, ME, USA) and housed in a conventional animal facility maintained at 20 ± 2°C and 50 ± 10% relative humidity with a 12 h light:12 h dark photoperiod. Food and water were provided *ad libitum*. Diet ingredients and feeding protocol have been previously described^7^. Briefly, upon arrival, the mice were acclimatized for one week and fed Purina Lab Chow #5001 (St. Louis, MO, USA). Subsequently, they were randomly separated into either CF (0.84% methionine w/w) or MR (0.12% methionine w/w) diets consisting of 14% kcal protein, 76% kcal carbohydrate, and 10% kcal fat (Research Diets, New Brunswick, NJ, USA) for the duration of the study. Young, old, and ApoE-KO mice were fed for 12 weeks while the acute and chronic treatments were 3 weeks and 36 weeks, respectively. Body weight and food consumption were monitored twice weekly for the duration of the study. For blood collection, animals were fasted for 4 h at the beginning of the light cycle to establish physiological baseline, and blood was collected from the retro-orbital plexus. Plasma was collected, flash frozen, and stored at −80°C until analyzed. Hearts were harvested, fixed in 10% formalin for histology or flash frozen, and stored at −80°C for further processing.

### Blood biochemical tests

Plasma amino acid analysis was performed with the Hitachi L-8800 HPLC amino acid analyzer (Bio Synthesis, Inc., Lewisville, TX, USA). The proteins were removed by treatment with 40 g/L sulfosalicylic acid/internal standard solution, separated by high-resolution ion-exchange chromatography, and then subjected to postcolumn ninhydrin derivatization; detection was conducted by spectrophotometry at 570 nm and 440 nm.

Total plasma homocysteine levels were measured by a competitive immunoassay using Immulite 1000 (Siemens Healthcare Diagnostics, Inc., Plainfield, IN, USA). Enzyme-linked immunosorbent assay (ELISA) kits were used to detect adiponectin (R&D Systems, Minneapolis, MN, USA) and FGF21 (Millipore Corp., Billerica, MA, USA).

### Histological analysis

Tissue processing for histology was provided by the Herbert Irving Cancer Center at Columbia University (New York, NY, USA). For H&E staining, heart tissue samples were fixed in a 10% formalin solution (Thermo Scientific, Waltham, MA, USA) and embedded in paraffin to obtain 5-μm-thick sections. Immunohistochemistry analysis was performed using antibodies for Ki67 (Abcam, Inc., Cambridge, MA, USA). Image analysis was conducted by HistoWiz, Inc. (Brooklyn, NY, USA) using ImagePro software (Media Cybernetics, Rockville, MD, USA). Three microscopic fields from each sample were photographed at 40× magnification.

### Molecular profile characterization

Total heart RNA was isolated using Qiagen RNA extraction kits (Gaithersburg, MD, USA); concentration and quality were assessed using the NanoDrop ND1000 (Wilmington, DE, USA). For the quantification of the target gene mRNA levels, reverse transcription-PCR was performed using a Perkin-Elmer GeneAmp PCR System 9600 with a High-Capacity cDNA Reverse Transcription Kit (Life Technologies, Carlsbad, CA, USA) as previously described^57^. Quantitations were conducted with StepOnePlus Real-Time PCR System using commercially available TaqMan primer-probe sets (Life Technologies, Carlsbad, CA, USA): *Nppa* (Mm01255747_g1), *Nppb* (Mm01255770_g1). *Prkaa1* (Mm01296700_m1), *Glut4* (Mm01245502_m1), *Cd36* (Mm01135198_m1), *Ppara* (Mm00440939_m1), *Pgc1a* (Mm01208835_m1), *Pparg* (Mm01184322_m1), *Adipoq* (Mm00456425_m1), *Adipor1* (Mm01291334_mH), *Adipor2* (Mm01184032_m1), *Fgf21* (Mm00840165_g1), and *Fgfr1* (Mm00438930_m1). Gene expression levels were assessed by the comparative CT (ΔΔCT) method with β-actin (NM_007393.1) as the reference gene.

Gene set enrichment analysis was performed by Phalanx Biotech (San Diego, CA, USA) based on previously described methods^58^,^59^ using the Kyoto Encyclopedia of Genes and Genomes database^60^. Briefly, total heart RNA was isolated using Qiagen RNA extraction kits, and the concentration and quality were assessed using the NanoDrop ND1000 and Agilent RNA 6000 Nano Assay. The OD260/280 of the samples was ≥1.99, and the average RNA integrity number was 7.4 ± 0.5. One-color Cy3 RNA labeling and array hybridization were used in the Agilent SurePrint G3 8x60K Mouse Gene Expression Arrays (Agilent Technologies, Santa Clara, CA, USA). Gene sets with absolute changes that increased or decreased by ≥1.5-fold with *P*-values < 0.001 and false discovery rates of *q* < 0.01 were selected for analysis.

### Non-invasive ECG in conscious mice

Mice were subjected to non-invasive ECGs to measure cardiac function as previously described^21^,^22^. Briefly, mice were gently removed from their cages and carefully positioned on an ECGenie recording platform (Mouse Specifics, Inc., Quincy, MA, USA). The platform floor electrodes were configured to passively contact three paws and provide ECG signals equivalent to Einthoven limb leads. To minimize stress, the mice were acclimatized on the platform for ~10 minutes before ECGs were recorded. Data from continuous recordings of 20–30 signals were used for analysis using an e-MOUSE software (Mouse Specifics). To determine the effects of the diets following β-adrenergic stimulation, the mice were administered intraperitoneal injections of isoproterenol (Sigma Aldrich, St. Louis, MO, USA) at 2.5 μg/g body weight twice daily for 3 days; on the fourth day, they were subjected to non-invasive ECGs as described above.

### Retrograde isolated heart perfusion analysis

After 14 weeks of dietary treatment, hearts were isolated from the old mice and perfused as previously described^61^. Briefly, mice were lightly anesthetized by isoflurane and euthanized by cervical dislocation. The hearts were then quickly removed and placed in ice-cold buffer followed by aortic cannulation for retrograde perfusion with phosphate-free Krebs-Henseleit buffer (Sigma-Aldrich, K3753) that was supplemented with calcium chloride and sodium bicarbonate maintained at 32°C. Cardiac function was measured using a balloon placed in the left ventricle, monitored using a pressure transducer, and analyzed using the EverBeat system acquisition software (Mouse Specifics)^46^,^62^. Cardiac intracellular Ca^2+^ regulation was assessed after a 10-minute initial data acquisition period by the infusion of Ca^2+^ into the solution at concentrations of 1 mM, 1.5 mM, 2 mM, and 2.5 mM. Cardiac contractility was tested by infusion of 100 nM of isoproterenol into the solution.

### Statistical analyses

Data are presented as the mean ± standard deviation (SD). Comparisons between two groups were conducted using a one-way or two-way analysis of variance (ANOVA) with Bonferroni post-tests for time course studies or Student's unpaired *t*-tests for end point analyses. All the analyses were performed using Prism 6 (GraphPad Software, La Jolla, CA, USA).

## Author Contributions

G.A. designed, performed the experiments, and wrote the manuscript; A.O. and D.C. assisted with animal experiments and analysis of samples. T.H. performed the mouse ECG and retrograde isolated heart perfusion experiments. F.P. conducted immunoassays; I.A. performed the amino acid analysis. D.O. reviewed the manuscript and verified the data.

## Supplementary Material

Supplementary Information Supplementary Information

## Figures and Tables

**Table 1:** Isolated heart retrograde perfusion tests at baseline and after calcium (Ca^2+^) and isoproterenol stimulation of old mice fed CF and MR diets

Diet	Treatment	HR (bpm)	Mean PSP (mm Hg)	Mean EDP (mm Hg)	DevP (mm Hg)	dP/dt (max) (mm Hg)	dP/dt (min) (mm Hg)
CF	Baseline	215 ± 76	42.3 ± 12.7	4.6 ± 5.6	37.7 ± 10.3	694.7 ± 180.3	(−) 459.5 ± 123.0
	Ca^2+^ (1 mM)	208 ± 84	8.5 ± 6.8*	5.5 ± 7.0	2.8 ± 1.5*	71.1 ± 19.6*	(−) 59.8 ± 3.8*
	Ca^2+^ (1.5 mM)	179 ± 87	12.8 ± 8.7*	3.1 ± 7.0	9.6 ± 1.9*	199.5 ± 50.3*	(−) 185.5 ± 123.9†
	Ca^2+^ (2 mM)	238 ± 62	23.5 ± 12.4†	0.1 ± 4.8	23.3 ± 9.7†	487.0 ± 198.5	(−) 484.6 ± 307.9
	Ca^2+^ (2.5 mM)	217 ± 40	22.2 ± 13.1†	(−) 1.1 ± 5.0	23.3 ± 10.6†	461.1 ± 204.4	(−) 312.1 ± 145.1
	Isoproterenol (100 nM)	219 ± 76	35.9 ± 14.2	0.1 ± 4.7	35.8 ± 12.5	663.5 ± 216.2	(−) 467.5 ± 172.8
MR	Baseline	225 ± 47	41.3 ± 16.5	8.2 ± 8.3	33.1 ± 12.3	652.1 ± 281.6	(−) 424.1 ± 143.5
	Ca^2+^ (1 mM)	223 ± 23	11.7 ± 9.3*	9.8 ± 9.6	1.9 ± 0.8*	57.7 ± 1.4*	(−) 79.9 ± 48.5‡
	Ca^2+^ (1.5 mM)	226 ± 34	13.1 ± 6.2*	5.1 ± 7.4	7.9 ± 2.2*	168.2 ± 35.3*	(−) 119.1 ± 26.6‡
	Ca^2+^ (2 mM)	223 ± 25	17.7 ± 5.9‡	2.2 ± 6.2	15.4 ± 5.9‡	382.6 ± 100.5†	(−) 186.5 ± 61.1†
	Ca^2+^ (2.5 mM)	219 ± 43	20.0 ± 8.5†	0.9 ± 6.3	19.9 ± 9.7†	460.3 ± 99.2	(−) 278.6 ± 128.8
	Isoproterenol (100 nM)	234 ± 54	43.6 ± 24.5	1.4 ± 4.5	42.2 ± 20.6	830.5 ± 487.9	(−) 552.0 ± 291.9

Statistical analysis conducted using one-way ANOVA followed by Bonferroni post-tests (n = 5 per group, ^†^P < 0.05, ^‡^P < 0.01, *P < 0.001). Abbreviations: heart rate (HR), peak systolic pressure (mean PSP), end diastolic pressure (mean EDP), left ventricular developed systolic pressure (devP), myocardial contractility (dP/dT_max_), and diastolic relaxation (dP/dT_min_).
