# Supplementary material for: Dietary Methionine Restriction in Mice Elicits an Adaptive Cardiovascular Response to Hyperhomocysteinemia
Source: Sci Rep. 2015 Mar 6;5:8886. doi: 10.1038/srep08886 (PMC4351514; doi:10.1038/srep08886)
Supplement: Supplementary Information [file srep08886-s1.pdf]

Dietary Methionine Restriction in Mice Elicits an Adaptive Cardiovascular Response to Hyperhomocysteinemia

Gene P. Ables[1*], Amadou Ouattara[1], Thomas G. Hampton[2], Diana Cooke[1], Franz Perrodin[1], Ines Augie[1] and David S. Orentreich[1]

[1]The Orentreich Foundation for the Advancement of Science, Cold Spring-on-Hudson, NY,
[2]Mouse Specifics, Inc., Quincy, MA

*Corresponding author: Gene P. Ables, PhD
The Orentreich Foundation for the Advancement of Science, Inc.
855 Route 301, Cold Spring, NY 10516
E-mail: gables@orentreich.org
Phone: (845) 265-4200
Fax: (845) 265-2410

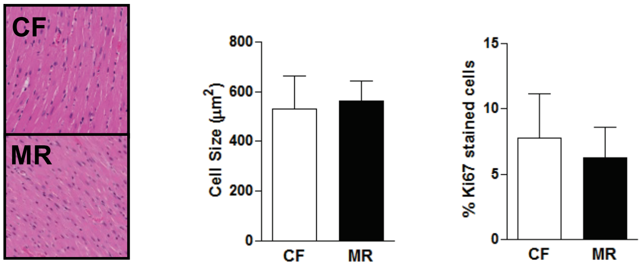

Figure I. A. Representative H&E stained heart tissues from young mice after 12 weeks of feeding. **B.** Cardiomyocyte sizes from heart tissues and **C.** immunohistochemistry staining for Ki67 of heart tissues as determine by image analysis described in the methods section. Statistics were measured by Student's unpaired *t*-test (n = 7 - 8 per group).

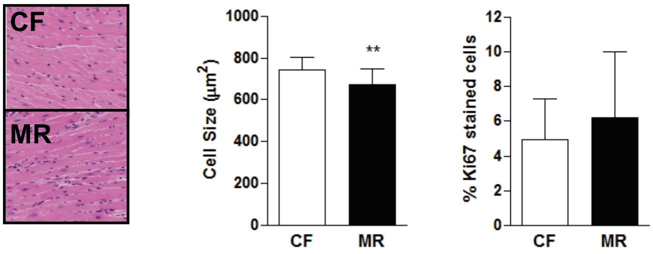

Figure II. A. Representative H&E stained heart tissues from old mice after 14 weeks of feeding. **B.** Cardiomyocyte sizes from heart tissues and **C.** immunohistochemistry staining for Ki67 of heart tissues as determine by image analysis described in the methods section. Statistics were measured by Student's unpaired *t*-test (n = 7 per group, $^{**}P < 0.01$).

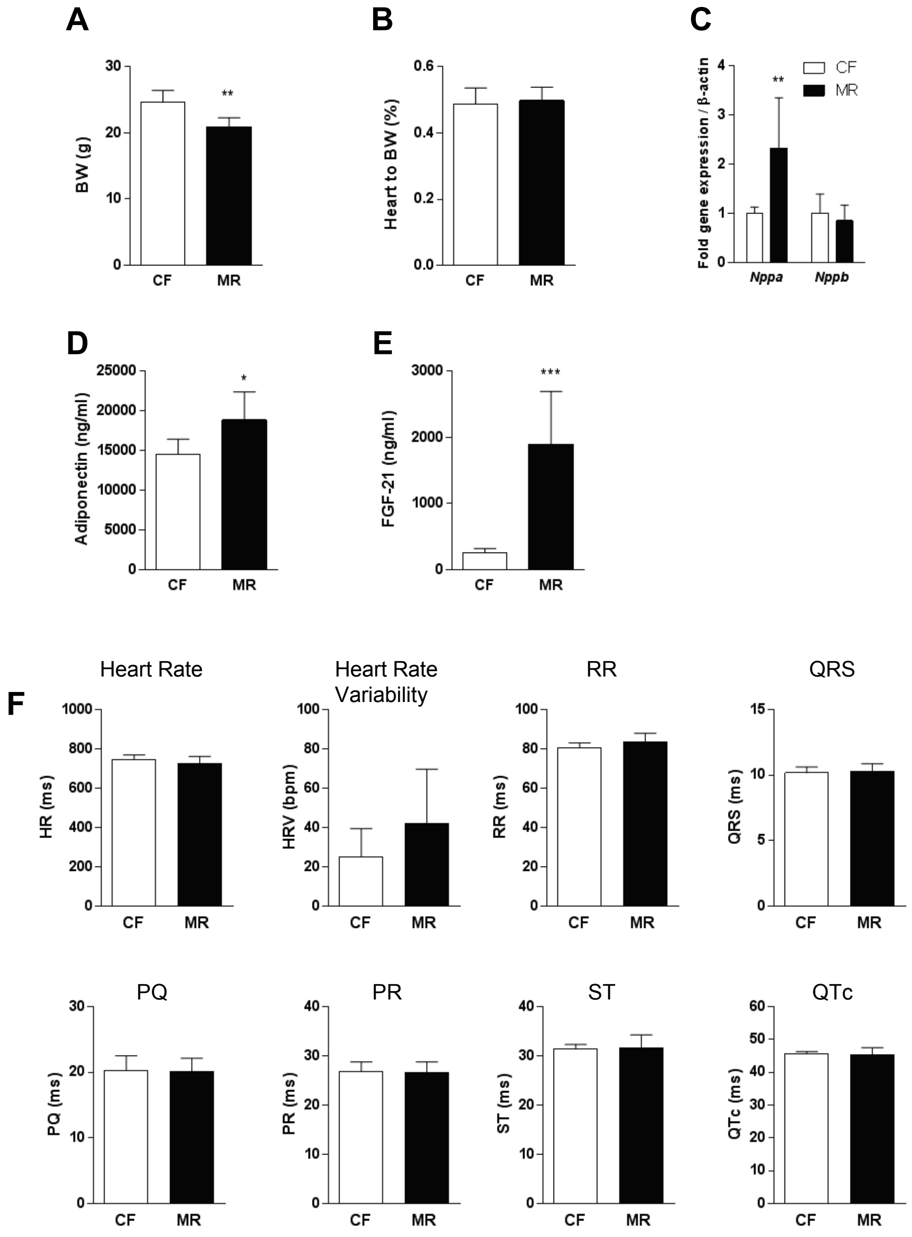

Figure III. Acute (3 weeks) diet treatment of 8 week old male C57BL/6J mice given control-fed (CF, 0.84% Met) and methionine restricted (MR, 0.12% Met) (n = 8 per group) diets. Body weights were lower in MR (**A**) while heart-to-body weight ratio was higher (**B**) compared to CF. Cardiac gene expression of *Nppa*, but not *Nppb*, was higher in MR compared to CF mice (**C**). Plasma adiponectin and FGF21 hormones were higher in MR mice compared to CF (**D** and **E**). Non-invasive ECG in conscious mice showed similar cardiac measurements in both groups (**F**). Statistics were conducted using Student's unpaired t-test ($^{*}P < 0.05$, $^{**}P < 0.01$, $^{***}P < 0.001$).

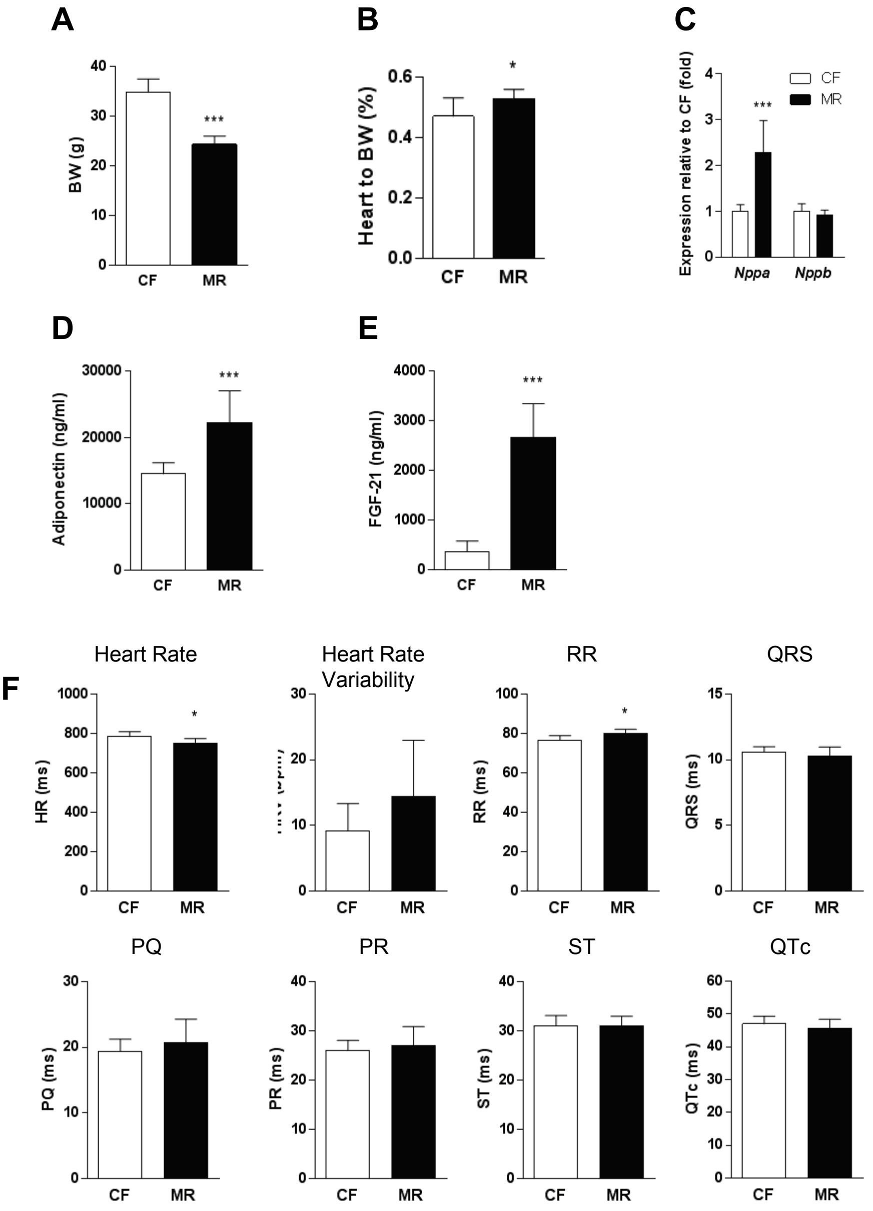

Figure IV. Chronic (36 weeks) diet treatment of 8 week old male C57BL/6J mice given control-fed (CF, 0.84% Met) and methionine restricted (MR, 0.12% Met) (n = 8 per group) diets. Body weights were lower in MR (**A**) while heart-to-body weight ratio was higher (**B**) compared to CF. Cardiac gene expression of *Nppa*, but not *Nppb*, was higher in MR compared to CF mice (**C**). Plasma adiponectin and FGF21 hormones were higher in MR mice compared to CF (**D** and **E**). Non-invasive ECG in conscious mice showed lower heart rate (HR) which corresponded with longer RR segment in MR mice compared to CF (**F**). Statistics were conducted using Student's unpaired t-test ($^{*}P < 0.05$, $^{**}P < 0.01$, $^{***}P < 0.001$).

Table I. Genes identified in the pathways regulated in cardiac tissues from CF and MR mice.

Genes in the Upregulated Pathways

Acacb	Acetyl-Coenzyme A carboxylase beta[1,2]
Adcy5	Adenylate cyclase 5[3,4]
Adcy6	Adenylate cyclase 6[3,4]
Adipor1	Adiponectin receptor 1[1]
Akt1	v-akt murine thymoma viral oncogene homolog 1[1,2]
Akt2	v-akt murine thymoma viral oncogene homolog 2[1,2]
Arhgef1	Rho guanine nucleotide exchange factor (GEF) 1[4]
Ednra	Endothelin receptor type A[4]
Exoc7	Exocyst complex component 7[1]
Gmpr	Guanosine monophosphate reductase[3]
Gys1	Glycogen synthase 1 (muscle) [2]
Insr	Insulin receptor[2]
Irs1	Insulin receptor substrate 1[1,2]
Irs2	Insulin receptor substrate 2[1,2]
Nme1	Non-metastatic cells 1[3]
Nme3	Non-metastatic cells 3[3]
Phkg2	Phosphorylase kinase, gamma 2 (testis) [2]
Pik3r2	Phosphoinositide-3-kinase, regulatory subunit 2 (p85 beta) [2]
Pla2g12a	Phospholipase A2, group XIIA [2]
Pold2	Polymerase (DNA directed), delta 2, regulatory subunit 50kDa[3]
Polr2c	Polymerase (RNA) II (DNA directed) polypeptide C, 33kDa[3]
Polr2e	Polymerase (RNA) II (DNA directed) polypeptide E, 25kDa[3]
Prkab1	Protein kinase, AMP-activated, beta 1 non-catalytic subunit[1,2]
Ramp3	Receptor (calcitonin) activity modifying protein 3[4]
Slc2a1	Solute carrier family 2 (facilitated glucose transporter), member 1[1]
Tnfrsf1a	Tumor necrosis factor receptor superfamily, member 1A[3]

Genes in the Downregulated Pathways

Acaa2	Acetyl-Coenzyme A acyltransferase 2
Actg1	Actin, gamma 1[5,6,8]
Cacna1s	Calcium channel, voltage-dependent, L type, alpha 1S subunit[5,6,8,10]
Cpt2	Carnitine palmitoyltransferase II[6,9]
Dbi	Diazepam binding inhibitor[9]
Hadh	Hydroxyacyl-Coenzyme A dehydrogenase[7]

Itga6	Integrin, alpha 6[5,6,8]
Itgb6	Integrin, beta 6[5,6,8]
Me1	Malic enzyme 1, NADP(+)-dependent, cytosolic[9]

Pathways affected in MR mice relative to CF mice as described in the methods and results sections:[1]adipocytokine signaling, [2]insulin signaling, [3]type 2 diabetes mellitus, [4]vascular smooth muscle contraction, [5]arrhythmogenic cardiomyopathy, [6]hypertrophic cardiomyopathy, [7]fatty acid metabolism, [8]dilated cardiomyopathy, [9]PPAR signaling, [10]cardiac muscle contraction.

Table 2 Formula of MR (CF) diets used in the study.

Ingredients	g / 100 g
L-Arginine	1.09
L-Histidine	0.32
L-Isoleucine	0.80
L-Leucine	1.08
L-Lysine	1.40
DL-Methionine	0.12 (0.84)
L-Phenylalanine	1.13
L-Threonine	0.80
L-Tryptophan	0.17
L-Valine	0.80
L-Glutamic Acid	3.34 (2.62)
Glycine	2.26
Corn Starch	53.38
Dextrose	4.86
Sucrose	14.57
Cellulose	4.86
Corn Oil	4.47
Minerals	3.40
Vitamins	0.97
Choline Bitartrate	0.19
Total	100.00
